# A Narrow Endemic or a Species Showing Disjunct Distribution? Studies on *Meehania montis-koyae* Ohwi (Lamiaceae)

**DOI:** 10.3390/plants9091159

**Published:** 2020-09-08

**Authors:** Atsuko Takano, Shota Sakaguchi, Pan Li, Ayumi Matsuo, Yoshihisa Suyama, Guo-Hua Xia, Xi Liu, Yuji Isagi

**Affiliations:** 1 Museum of Nature and Human Activities, Hyogo. 6 Chome, Yayoigaoka, Sanda, Hyogo 669-1546, Japan; 2 Graduate School of Human and Environmental Studies, Kyoto University, Yoshida-nihonmatsu-cho, Sakyo-ku, Kyoto 606-8501, Japan; sakaguci54l@gmail.com; 3 College of Life Sciences, Zhejiang University, 866 Yuhangtang Rd., Xihu District, Hangzhou 310058, China; panli_zju@126.com; 4 Tohoku University, Kawatabi Field Science Center, Graduate School of Agricultural Science, 232-3 Yomogida, Naruko-onsen, Osaki, Miyagi 989-6711, Japan; ayumi.matsuo.b2@tohoku.ac.jp (A.M.); yoshihisa.suyama.e2@tohoku.ac.jp (Y.S.); 5 School of Forestry and Bio-Technology, Zhejiang A & F University, No. 666 Wusu Str., Lin’an District, Hangzhou 311314, China; ligy1956@163.com; 6 Wuyanling National Nature Reserve of Zhejiang, Northwest of the County, Wenzhou 325500, China; liuxiliushi@163.com; 7 Division of Forest and Biomaterials Science, Faculty of Agriculture, Kyoto University, Kitashirakawa-oiwake-cho, Sakyo-ku, Kyoto 606-8502, Japan; isagiy@kais.kyoto-u.ac.jp

**Keywords:** Lamiaceae, *Meehania montis-koyae*, MIG-seq, population genetic analysis, Sino-Japanese disjunct distribution

## Abstract

*Meehania montis-koyae* Ohwi (Lamiaceae), which has been considered a narrow endemic and endangered species in Japan, was found in eastern China in 2011. China and Japan belong to the same floristic region and share many plant species, but it is very rare that Japanese narrow endemic species are newly found outside of the country. We examined herbarium specimens of both countries, and conducted analyses of molecular phylogenetics, population genetics, and divergence time estimation using two nuclear (ITS and ETS) gene regions and MIG-seq data. Chinese plants tend to become larger than Japanese, and they are different in leaf shape and floral features. Molecular phylogenetic analysis shows Chinese and Japanese *M. montis-koyae* are the closest relatives to each other. Population genetic analysis indicates no current gene flow between the Chinese and Japanese populations, and divergence time analysis shows they were separated during the late Miocene. We reach the conclusion that Chinese and Japanese *M. montis-koyae* have already become distinct biological entities, and a new taxon name *Meehania zheminensis* A. Takano, Pan Li, G.-H.Xia is proposed for the Chinese plants. A key to Asian *Meehania* species is provided.

## 1. Introduction

The Sino-Japanese floristic region is well known for its richness in species and high degree of endemism [1,2]. The floristic relationships between eastern China and Japan are closer than that between northeastern China and Korea; the similarity indices of genera and species of seed plants in the two regions are 72.6% and 43.3%, respectively [3]. Many plants in eastern China are conspecific with those in Japan, e.g., *Cercidiphyllum japonicum* Siebold et Zucc. ex Hoffm. et Schult., *Nandina domestica* Thunb., *Disanthus cercidifolius* Maxim., *Kirengeshoma palmata* Yatabe. In some corresponding species, such as *Cryptomeria japonica* (in Japan) and *C. fortunei* (in East China), whether they should be treated as distinct or merged into a single species is still in question [3].

The genus *Meehania* Britton is a small herbaceous genus in Lamiaceae. It contains less than ten species [4,5]; only one species, *M. cordata* (Nutt.) Britton, is distributed in eastern North America, and the others in East Asia, showing a typical pattern of Arcto-Tertiary floristic disjunction [6,7,8]. Deng et al. (2015) conducted a biogeographic analysis of the genus and showed that *Meehania* is monophyletic in the tribe Menteae [9]. *Meehania cordata*, the sole North American species, is sister to a clade containing the eastern Asian species. Divergence between *M. cordata* and the eastern Asian clade occurred about at 9.81 Mya according to the Bayesian relaxed clock methods applied to the combined nuclear DNA sequence data. These latter authors showed an Arcto-Tertiary origin of *Meehania*, its present distribution being a result of vicariance and southward migrations of populations during climatic oscillations in the middle Miocene with subsequent migration into eastern North America via the Bering land bridge in the late Miocene.

*Meehania montis-koyae* Ohwi has been thought to be a narrow endemic to Japan [4,10,11]. The species was first described in 1933 based on a specimen collected from Mt. Koya, Wakayama Prefecture, western Japan. However, no other population besides that of the type locality was been found in the prefecture (A. Naitou, Wakayama Prefectural Museum of Natural History, pers. comm.), and the current distribution is only known from the Hyogo Prefecture. Extant populations are less than ten, and all are small (several hundred shoots at the largest population), threatened, and distributed within a small area of western Hyogo. The plant has been included in the National Red List of Threatened Plants of Japan (Ministry of Environment: http://www.env.go.jp/press/files/jp/110615.pdf) as a Threatened II category. Surprisingly, however, the species was recently found in Zhejiang and Fujian provinces in China [12]. The floras of eastern China and Japan are similar, but it is rare that a Japanese narrow endemic and endangered species is newly found in China.

Do Chinese *M. montis-koyae* really belong to the same biological entity as the Japanese ones? Or are they similar but represent a distinct taxon? If there were significant morphological and genetic differences, and no genetic admixture between the plants of both countries was found, we could treat both as distinct taxa. To answer this question, we conducted molecular phylogenetic analyses, divergence time estimates, and performed population genetic and morphological comparisons.

## 2. Results

### 2.1. Morphological Differences between Chinese and Japanese M. montis-koyae

**Figure 1:**
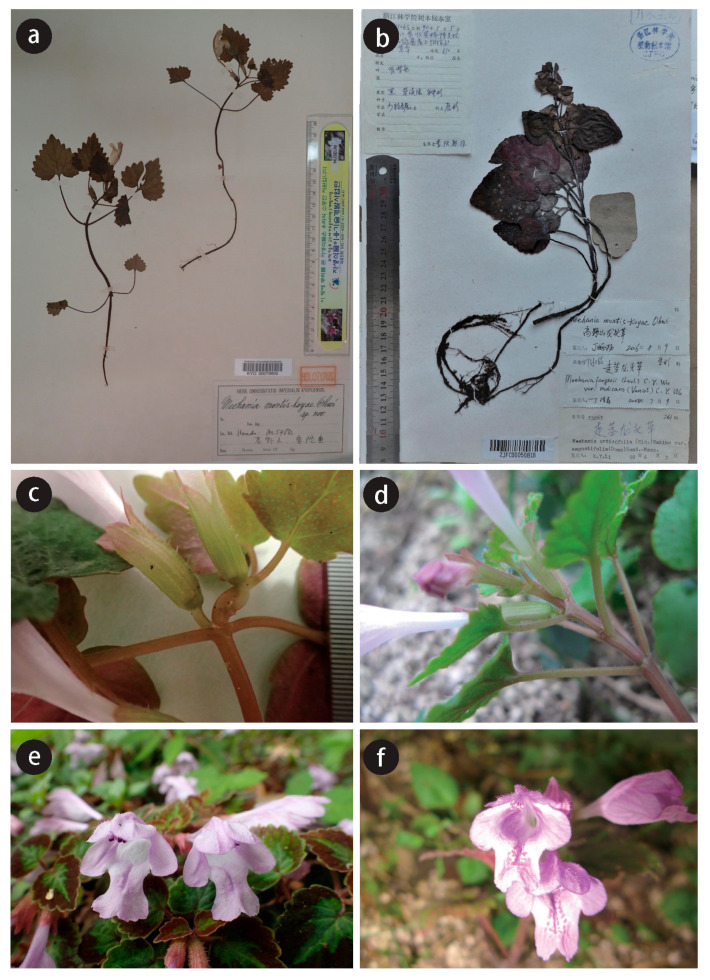
Chinese and Japanese *M. montis-koyae*. (**a**) Holotype of *M. montis-koyae* (KYO), collected from Mt. Koya, Wakayama Pref., Japan; (**b**) Specimen collected from Taishun, Zhejiang, China (ZJFC00050818); (**c**,**d**) close-up of stem: Japanese plant (**c**), Chinese plant (**d**); (**e**,**f**) flowers: Japanese (**e**), Chinese plant (**f**).

There were several morphological differences between the Chinese and Japanese plants of *M. montis-koyae* (Figure 1). Chinese plants are larger in size and robust compared to Japanese ones (Figure 1a,b). The stem is also robust and four-angled in Chinese plants, but narrow and rounded in Japanese plants (Figure 1c,d). Leaf blade is thin, deltoid, with a crenate or roundly dentate margin in Japan, however, it is rather thick, cordate to obtuse with a (sometimes shallowly) crenate margin in China (Figure 1a,b). The size of the largest leaf in a shoot is significantly larger in Chinese plants (blade length 4.4 ± 1.2 cm, width 4.0 ± 0.9 cm, *n* = 10) than in the Japanese specimens (blade length 3.1 ± 0.8 cm, width 2.8 ± 0.6 cm, *n* = 21; two-tailed *t*-test, *p* < 0.01). Chinese plants have more flowers per shoot (5.7 ± 2.4, *n* = 11) than Japanese plants (3.2 ± 1.3, *n* = 12; two-tailed *t*-test, *p* < 0.01). Examining images of *M. montis-koyae* from China and Japan, we always found red spots on the center lobes of the lower labellum in Chinese plants, but no such spots in the Japanese ones (Figure 1e,f). Further, we noticed that the flowering season is longer in China than in Japan. The date when flowering specimens were collected or photos were taken in China is from March (19 is the earliest) to May (15 is the latest). In Japan, the earliest day of flowering specimen was 18 April, and the latest date was 20 May. Altitudinal differences were also found. Chinese *M. montis-koyae* are found from the forest floor at altitudes of 450–1600 m, but those of Japanese populations are established at the margin of deciduous forests along a streamlet, often on scree slopes from 200–480 m. 

The Chinese *M. montis-koyae* appear to be evergreen perennials, since many sterile specimens of the species were deposited in Chinese herbaria (e.g., ZMNH0031633, 0031634, 0031635 were collected on 15 August 1985; ZMNH0031632, 0031637 on 17 August 1987; ZMNH0031639 on 21 July 1971; ZJFC00050817 on 17 November 1986). In Japan, *M. montis-koyae* is a perennial herb with a unique life cycle: both fertile and sterile shoots disappear from the ground surface until late June (after fruiting), and new shoots mainly emerge in late fall to early winter [13]. The earliest collection date of Japanese *M. montis-koyae* was 11 February (C2-009265, HYO), and the latest date was 27 July (C2-009287, HYO). According to previous reports, the plants emerge in late autumn to winter [13], but they are too small to be found accidentally; only collectors knowing the exact location were able to collect specimens. However, in cultivated Chinese plants it was found that shoots also disappeared after fruiting (Li, unpublished data), so more phenological studies in the field are necessary for the Chinese *M. montis-koyae*.

### 2.2. Maximum Likelihood Analysis by Using nrDNA and MIG-seq Data

**Figure 2:**
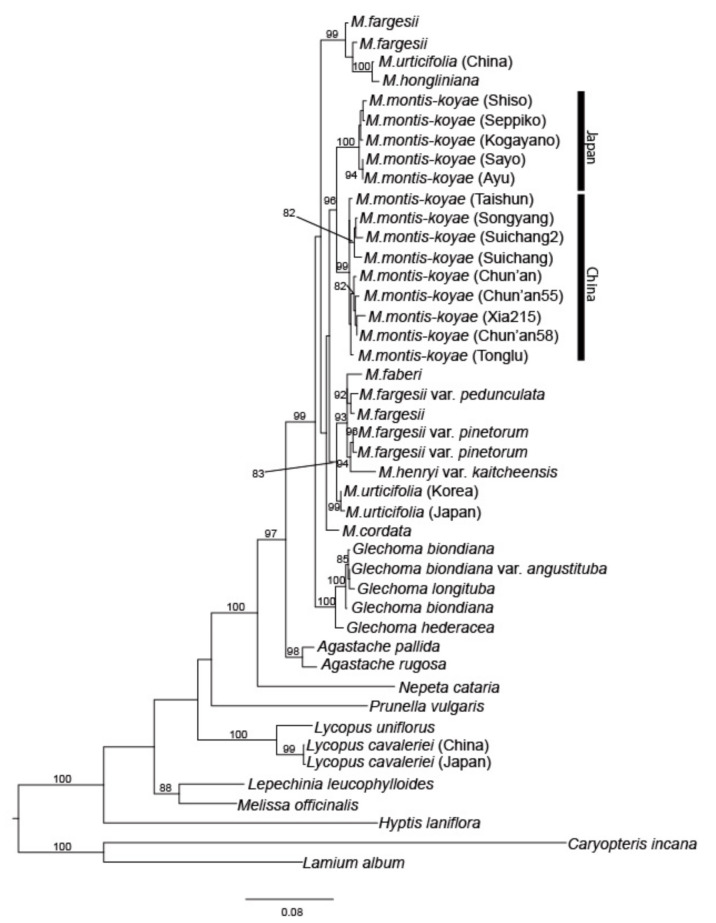
Maximum Likelihood (ML) tree using nuclear DNA (ITS + ETS). Numbers below/above branches indicate bootstrap values (*n* = 1000).

In total, 1117 bp of a combined nrDNA data matrix were used (Figure 2). Monophyly of *M. montis-koyae* is highly supported (bootstrap support, BS = 94%), and the Chinese and Japanese plants each formed a well-supported subclade (BS = 99% for the former, BS = 100% for the latter). Interestingly, *M. urticifolia* is apparently polyphyletic: Japanese and Korean plants formed a well-supported subclade (BS = 96%) sister to *M. cordata* and *M. montis-koyae*, though credibility of the clade was weak, while Chinese *M. urticifolia* formed a subclade with *M. hongliniana* B.Y. Ding and X.F. Jin, appearing as sister to *M. fargesii* with high BS support (95%). 

**Figure 3:**
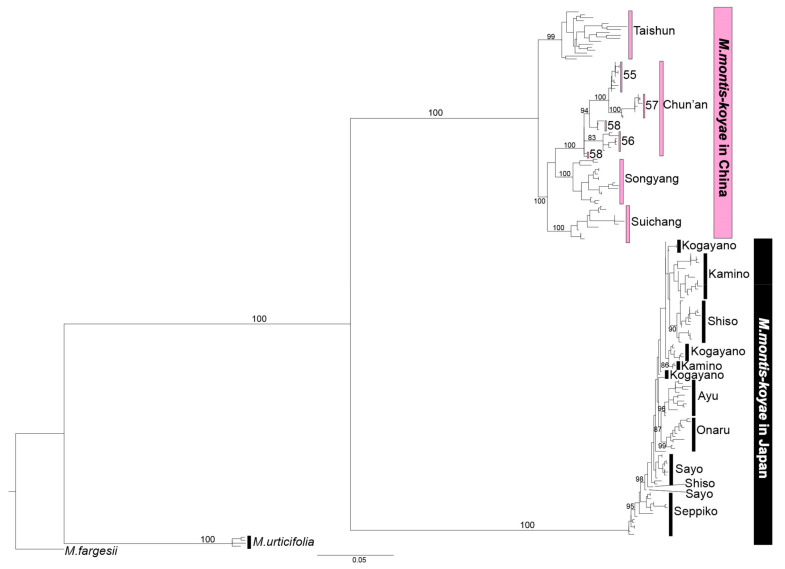
Maximum likelihood tree inferred from MIG-seq dataset, estimated by IQ-TREE 1.6.12. Numbers above branches are bootstrap values (BS, >80% only) generated from 1000 replicates.

Maximum Likelihood (ML) analysis using Multiplexed ISSR genotyping by sequencing (MIG-seq) data also showed that each Chinese and Japanese plant formed a well-supported subclade (BS = 100% for the former, 100% for the latter) (Figure 3). In the Japanese subclade, plants in the same population formed a cluster, except for the Kamino, Kogayano, Sayo and Shiso populations, but support for the clusters was low. On the other hand, the Chinese populations can be clearly divided into four subclades according to their geographical distribution: Taishun (BS = 99%), Suichang (BS = 100%), Chun’an (LP174755-58, BS = 100%), and Songyang (BS = 100%).

### 2.3. Population Analysis Using MIG-seq Data

**Figure 4:**
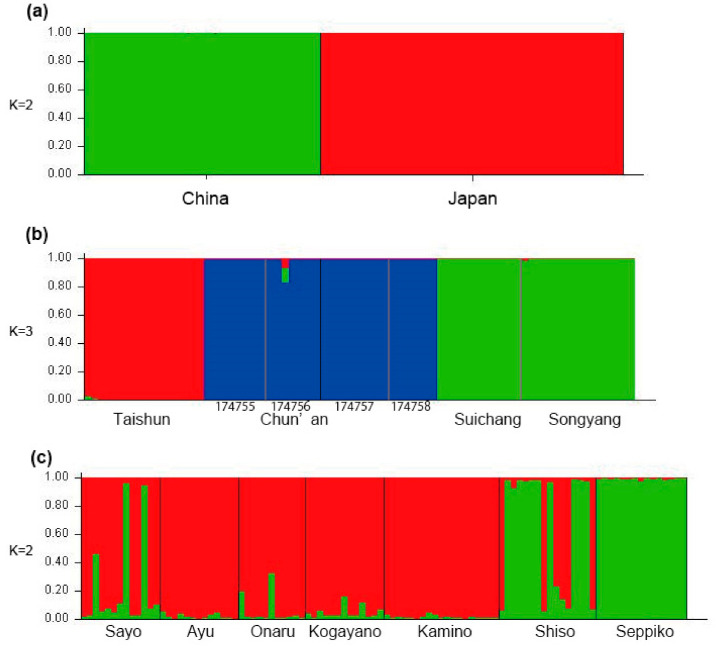
Results from a Bayesian population assignment test with STRUCTURE for *Meehania montis-koyae* based on 338 MIG-seq SNPs. (**a**) Chinese and Japanese individuals (*n* = 178), (**b**) Japanese individuals only (*n* = 100), (**c**) Chinese individuals only (*n* = 78).

The genetic structure estimated by Bayesian clustering analysis supported *K* = 2 as the optimal number of clusters for the datasets of all individuals, based on Δ*K* statistics. The optimal number of clusters for Japanese individuals only is *K* = 2, while that for Chinese individuals only is *K* = 3. The datasets of all individuals were clearly clustered in Chinese and Japanese plants, and no gene flow was detected in between (Figure 4a). Chinese populations were clearly split into three clusters corresponding to their geographic distributions—Chun’an (174755-58), Taishun, Songyang, and Suichang—and little genetic admixture is found among those clusters (Figure 4b). In the case of the dataset of the Japanese plants only, genetic structure was found between eastern (Seppiko + Shiso) and western subpopulations (the rests), but admixture of the two clusters was found among them (Figure 4c). The analysis of population genetic distances also clearly showed significant genetic distances between Chinese and Japanese populations (0.556–0.600, Table 1). Further, genetic distances found in the Chinese populations (0.406–0.432) are much larger than those within the Japanese populations (0.292–0.315). In both Chinese and Japanese populations, observed (*Ho*) and expected (*He*) heterozygosities, and Shannon’s information index (*I*) were very low (*Ho* = 0.004–0.012, *He* = 0.002–0.013, *I* = 0.004–0.02, Table 2).

### 2.4. Divergence Time Analysis of M. montis-koyae

**Figure 5:**
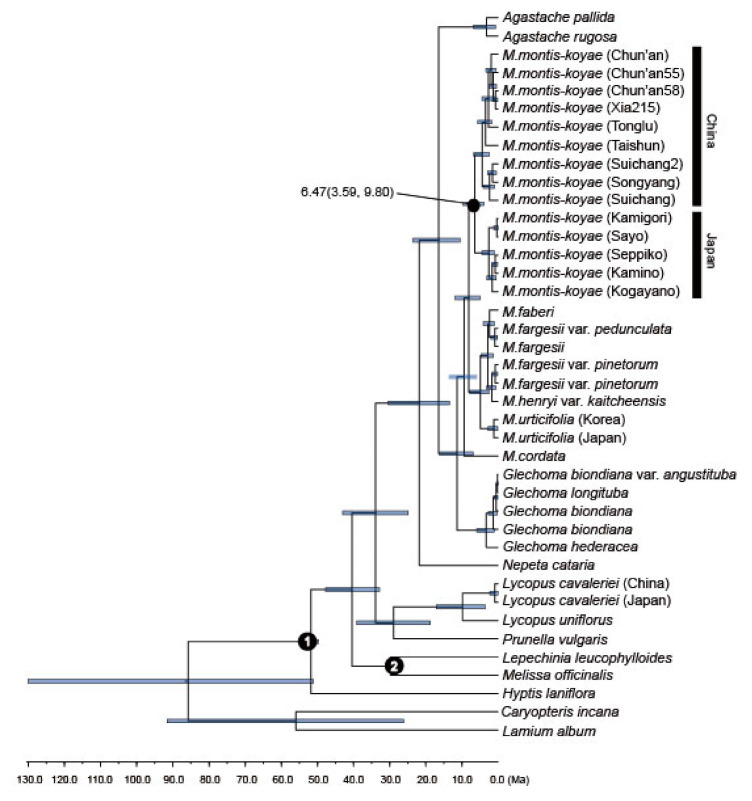
Results of the BEAST analysis based on combined nuclear (ITS and ETS) data. Gray bars represent the 95% highest posterior density intervals for node ages. Fossil calibrations are marked with numerals 1 and 2.

The chronogram and results of divergence-time estimation based on nrDNA are shown in Figure 5. The divergence time between Chinese and Japanese *M. montis-koyae* was estimated at 6.47 Ma, with 95% highest posterior probability (HPD) of 3.59–9.80 Ma, i.e., in the late Miocene.

## 3. Discussion

This study supported and complemented the hypothesis of [9] on the evolution of *Meehania*, by increasing samples of *M. montis-koyae* in China and Japan. Those authors suggested that the plausible ancestral area of *Meehania* may have been the high-latitude areas of Eurasia, and the decrease in annual mean temperature at high latitudes provided opportunities for biota dispersal and subdivision. The present distribution of *Meehania* in eastern North America and northeastern and southeastern Asia could result from the vicariance of south-migrating populations during climatic oscillation and further fragmentation and dispersal of these populations [9]. From our analyses, Chinese and Japanese *M. montis-koyae* might have diverged during the late Miocene (8.58 Ma, 95% HPD: 4.93–13.51) when the climate became cooler [14]. Not only in *M. urticifolia* and the other Chinese *Meehania* lineages mentioned by [9], but also in the *M. montis-koyae* lineage, there may be south-migrating populations.

Morphological and molecular analyses revealed that Chinese and Japanese *M. montis-koyae* are apparently the closest relatives to each other, but they have already become distinct biological entities. Morphologically, Chinese plants are larger in size and the number of flowers and leaves per shoot is also different. Ecologically, the Chinese plant is an evergreen perennial while the Japanese plant is perennial with a short dormancy period [13]. The flowering season is in spring for both, but it seems longer in China (late March to mid May) than in Japan (late April until late May). Molecular phylogenetic analyses using combined nrDNA and MIG-seq data showed that the two are close relatives (Figure 2), and that the plants from both countries are not admixed (Figure 3). Population genetic analyses using STRUCTURE also show no recent gene flow between the plants of the two countries (Figure 4a). 

After separation in the late Miocene, there may have been no possibility of gene flow between Chinese and Japanese *M.montis-koyae* populations. During the Quaternary period, the East China Sea (ECS) land bridge connected the forests of East China, South Japan, and probably the Korean Peninsula [15], at the last glacial maximum (LGM: c. 21,000 years before present [16]), when sea levels were c. 85–130 m below the present [17]. The ECS has acted as a corridor for the two-way migration of animals and plants, providing the possibility of secondary contact for disjunct populations or related species between China and Japan, e.g., [18,19,20,21]. The ECS land bridge was covered in a large part by temperate deciduous forest, but may have comprised local arid patches of nonforested vegetation (e.g., steppe, grasslands) [16]. Since *M. montis-koyae* favors margins of deciduous forests in lower elevations, and is often found growing along steamlets in high humidity, such arid environments could have been barriers for migration of the species.

In both Chinese and Japanese populations, the expected heterozygosity (*He*, 0.002–0.013), observed heterozygosity (*Ho*, 0.004–0.012), and Shannon’s information index (*I*, 0.004–0.02) were very low. In the case of Japanese *M. montis-koyae*, the plants can propagate asexually by subterranean shoots. They are basically entomophilic and self-compatible [22], and such low genetic diversity indices suggest that selfing or asexual reproduction is dominant in the species due to insufficient pollinator visits during the flowering season. Since no such ecological studies have been performed for Chinese populations, studies on phenology and breeding systems are still needed. On the other hand, the effect of genetic drift also has to be considered. In the Quaternary period, plant migration may have occurred corresponding to climatic fluctuations. Since *M. montis-koyae* is basically barochoric, bearing seeds with no special attachments such as arils or elaiosomes, long dispersal distance by seeds can not be expected. Local extinction and population fragmentation could likely have occurred with subsequent genetic drift. Present populations are often threatened by human activities such as construction along rivers or illegal digging for commercial usage. The low genetic variation found within *M. montis-koyae* may be a cause of either of these effects or their synergy.

Phylogenetic analyses with an increased number of taxa and plants have revealed some inconsistency in the current classification of *Meehania* species. Neither *M. fargesii* nor *M. urticifolia* seem to form single subclades. The former species has several infraspecific taxa, but each variety appears to belong to a different subclade. In case of *M. urticifolia*, a widely distributed species from Japan, Korea, and China, the Japanese and Korean plants formed a well-supported subclade (BS = 99, Figure 2), but the Chinese plants were shown to belong to a different clade and constitute a subclade with *M. hongliniana* B.Y. Ding and X.F. Jin. Thus, re-evaluation of the phylogenetic systematics of these taxa is needed.

In summary, Chinese and Japanese *M. montis-koyae* are morphologically, ecologically, and genetically different, and have already become distinct entities. Therefore, we propose a new name, *Meehania zheminensis*, for the plants of *M. montis-koyae* from China. As mentioned, *M. montis-koyae* was originally described based on a specimen collected from Japan, so the name should be applied to Japanese plants.

### 3.1. Taxonomic Treatment

#### *Meehania zheminensis* A. Takano, Pan Li & G.-H. Xia *sp. nov.* (浙闽龙头草)

**Figure 6:**
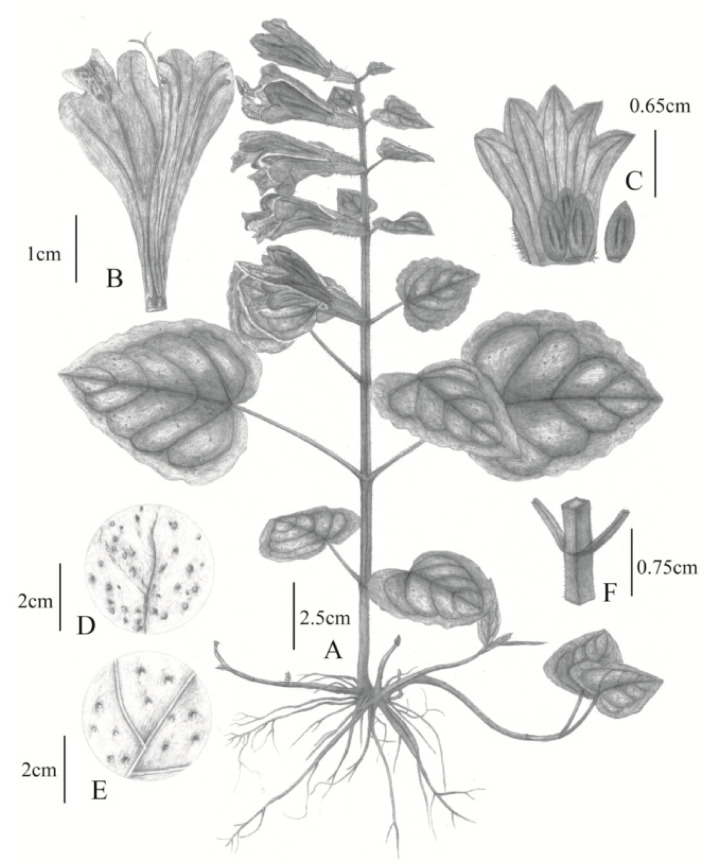
Illustration of *Meehania zheminensis* A. Takano, Pan Li and G.-H.Xia; (**A**) plant with inflorescence; (**B**) corolla dissected; (**C**) calyx dissected, with four seeds; (**D**) adaxial surface of leaves; (**E**) abaxial surface of leaves; (**F**) stem node. Drawn by Xinjie Jin.

TYPE: CHINA, Zhejiang Prov., Tonglu County, Baiyunyuan, *Pan Li*, *LP207976*, holotype (HZU), isotypes (HHBG, HYO, KYO, KUN, PE, TI, TNS, ZM)–Figure 1b,d,f and Figure 6.

Similar to *Meehania montis-koyae*, but with larger vegetative shoots with a four-angled stem up to 40-cm high (vs. round stem up to 20-cm high in *M. montis-koyae*), bearing reddish spots on the center lobe of lower labellum (vs. no such spots on the labellum), distributed in China.

Perennial herbs. Shoot erect, 10–40 cm in height. Stems tetramerous, slender and without stolons, puberulent throughout when young, and becoming rare or glabrous, densely hairy only on nodes. Leaves opposite, largest in the middle of shoot, chartaceous, petiole 0.5–5.0-cm long, become shorter toward apex shoot, sparsely hairy, blade cordate to reniform, 2.8–5.0-cm long, 2.0–4.5-cm-wide, base cordate, apex obtuse, shallowly crenate to undulate, glandular-dotted, scabrous on upper surface, sparsely pubescent on lower surface. Flowers March to June, solitary and axillary, opposite each other, 1 to 3 (to 7) pairs on upper stems. Bracteoles setaceous, 1–1.5-mm long. Pedicel 2–3-mm long, puberulent. Calyx tubular, bilabiate, 12–14mm long, 15-nerved, puberulent, gland-dotted; upper lip ca. 3-mm long, deeply bilobed, lobes deltoid acute. Corolla tubular, bilabiate, rose-purple, ca. 4-cm long; tube slender near base, abruptly dilated upward, glabrous; upper lip ca. 7-mm long, shallowly bilobed, lower lip ca. 10-mm long, trilobed, middle lobe largest, apex shallowly bilobed, bearing red spots in the center. Stamens 4, one pair of stamens bearing short filaments attached on the inside throat of the corolla, and the other pair with longer filaments attached on the bases of lateral corollas, filament glabrous, anther thecae with two locules, glabrous, pale purple. Style longer than corolla, stigma bilobed, pale red-purple, ovary four lobed, hairy. Nutlets narrowly ellipsoid, ca. 3-mm long, punctate-puberulent. Fruits April to July.

### 3.2. Other Specimens Examined

**CHINA. Zhejiang Province. TongluCounty**: Baiyunyuan, 9 April 1978, *L. Hong 78,579* (HHBG); *ibidem*, 15 May 2009, *G.-Y. Li* et al. *TL090501* (ZJFC); *ibidem*, 9 April 2014, *unknown collector 5429* (HHBG). **Kaihua** **County**: Mt. Gutian, 15 April 1984, *C.-F. Wu 0022* (ZJFC); Mt. Gutian, Baishukeng, 28 May 1990, *Y.-L. Xu* et al. *0743* (ZM); Mt. Gutian, Lukeng, 4 April 1985, *F.-G Zhang 107* (ZJFC). **Suichang** **County**: Mt. Jiulong, Daxikeng, 15 August 1985, *Zhejiang Museum of Natural History 4932* (ZM); *ibidem*, 22 May 1986, *F.-G. Zhang 5229* (ZM); Mt. Jiulong, 23 July 2017, *X.-L. Xie XXL170009* (HZU); Mt. Jiulong, on the way from Xikengkou to Huangjiping, 13 April 2020, *P. Li LP208014* (HZU). **Songyang** **County**: Ruoliao, 25 August 2017, *X.-L. Xie XXL170209* (HZU). **Longquan** **County**: Mt. Fengyang, 21 July 1972, *Medicinal Flora of Zhejiang 2633* (ZM); **Qingyuan County**: Mt. Baishanzu, on the way from Chegen village to Baipuxia, 20 May 2019, *P. Li LP196665* (HZU). **Taishun** **County**: Yangxi Forest Farm, 19 November 1986, *L.-H. Lou* et al. *0497* (ZJFC); Huangqiao village, Chenwukeng, 5 May 1990, *G.-Y. Li* et al. *TH065* (ZJFC); *ibidem*, 5 May 1990, *G.-Y. Li* et al. *691* (ZM); Wuyanling National Nature Reserve, 24 March 2017, *X. Liu LP173032* (HZU); *ibidem*, 10 April 2017, *F.-G. Zhang* et al. *234* (ZM); *ibidem*, 3 April 2020, *X. Liu LP208004* (HZU).

### 3.3. Key to the Species of Asian Meehania

1a Rhizomes stout, short, with slender decumbent leafy stolons after flowering; stems and petioles sparsely pilose; flowers 1 or 2 in leaf axils, forming 2- or 4-flowered verticillasters, generally 3 or 4 verticillasters forming a spike-like raceme, having no smell to the plant body-------------------------------------------------------------------------------------------------------------------------------- (2)
1b Rhizomes slender, elongate, without stolons; stems and petioles subdensely puberulent; flowers solitary and axillary, forming a pair, with 1 to 4 pairs on a stem, stink-bug-like smell to the plant body ------------------------------------------------------------------------------------------------------- (3)
2a Leaves ovate, long elliptic to narrowly lanceolate, base slightly cordate or cuneate--------------------------------------------------------------------------------------------------------------------------------------------- (4)
2b Leaves cordate to ovate, base cordate, rarely truncate to rounded ----------------------------------- (5)
3a 10–40 cm in height, bearing reddish spots on the lower labellum, distributed in China-------------------------------------------------------------------------------------------------- ***Meehania*** ***zheminensis*** **sp. nov.**
3b 10–20 cm in height, bearing no spots on the lower labellum, distributed in Japan-------------------------------------------------------------------------------------------------------------------------------*M. montis-koyae*
4a Leaves oblong-elliptic or ovate, 5.0–12.0 cm long, 2.0–5.0 cm wide ---------------------------------- (6)
4b Leaves narrowly lanceolate, 6.0–12.0 cm long, 1.2–2.5 cm wide ------------------------- *M. pinfaensis*
5a Verticillasters in terminal and lateral racemes; calyx tube narrowly tubular-------------- *M. henryi*
5b Verticillasters in terminal racemes or 2-flowered, inserted in leaf axils of upper 2 or 3 leaf pairs on stem; calyx campanulate or ± tubular --------------------------------------------------------------------- (7)
6a having dimorphic (=sterile and fertile) stems ---------------------------------------------- *M. hongliniana*
6b having monomorphic stems ---------------------------------------------------------------------------- *M. faberi*
7a Calyx campanulate, inconspicuously veined, floccose-villous on veins, teeth triangular, subequal -------------------------------------------------------------------------------------------------- *M. urticifolia*
7b Calyx narrow, ± tubular, conspicuously veined, sparsely pubescent on veins, teeth narrowly triangular ---------------------------------------------------------------------------------------------------- *M. fargesii*

## 4. Materials and Methods 

### 4.1. Herbarium Studies on Morphology, Phenology, and Habitats of M. montis-koyae

We examined specimens of *M. montis-koyae* in the following herbaria in China and Japan: Kyoto University (KYO), Herbarium of Hangzhou Botanical Garden (HHBG), Museum of Nature and Human Activities, Hyogo (HYO), Herbarium of Zhejiang University (HZU), Herbarium of Zhejiang University of Agriculture and Forestry (ZJFC), Zhejiang Museum of Natural History (ZM). We measured maximum leaf blade length and width of the largest leaf on a sheet, and counted number of leaves and flowers per shoot. We also took altitudinal information and collection dates if flowers were available on the sheet to determine the flowering period. Additionally, we searched websites and obtained photos of flowering *M. montis-koyae* from colleagues and checked the coloration of the flowers in China and Japan.

### 4.2. Sampling for Molecular Analyses

**Figure 7:**
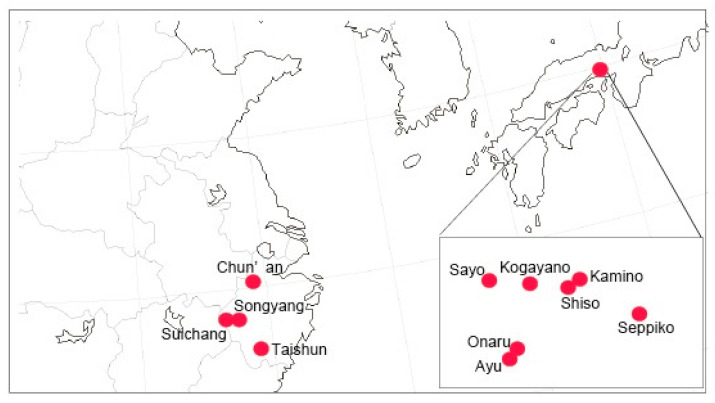
The study sites of *M. montis-koyae*.

For molecular analyses, we collected a few leaves from each 11 to 33 individual per population of 11 *M. montis-koyae* populations in both countries. Four populations from China were studied, though in Chun’an sampling was partitioned into four subpopulations (=174755, 174756, 174757, 174758). Every site was located along the trail, ca. 2 km apart from each other); seven populations are from Japan. Sampling sites are shown in Figure 7. To avoid illegal removal or damage to the endangered species, we refrain from describing details of the locality here.

### 4.3. DNA Extraction, Amplification, and Sequencing

Total genomic DNA was isolated from silica-gel dried or fresh leaf material using a modified CTAB method [23]. Two nuclear ribosomal regions (ITS and ETS) were selected for phylogenetic inference. ITS and ETS were amplified and sequenced using the primers ITS1 and ITS4 for ITS [24], and the primers ETS-bdf1 [25] and 18SE [26] for ETS. Amplified PCR products were run on 1.0% agarose gel in TAE buffer, and detected by Atlas Clear Sight DNA staining (Bioatlas, Estonia). The PCR products were then purified using Microspin S-300 HR Columns (GE Healthcare Japan, Tokyo, Japan), and used as templates for the sequence reactions. After purification following the manufacturer’s protocol, the samples were directly sequenced in both directions using an ABI 310 genetic analyzer (Applied Biosystems Japan, Tokyo, Japan).

### 4.4. MIG-seq Preparation

In total, 178 samples of *M. montis-koyae* were used, representing four populations in China and seven in Japan, with up to 33 samples per population. Additionally, one *Meehania fargesii* (H. Lév.) C.Y. Wu and four *Meehania urticifolia* (Miq.) Makino were selected as outgroups and analyzed. Multiplexed ISSR genotyping by sequencing (MIG-seq) was used for single-nucleotide polymorphism (SNP) detection [27]. Preparation of the MIG-seq library was performed under standard conditions according to [27]. Eight primers of the most recommended set of MIG-seq primers (set-1) [27] were employed for the 1st PCR. PCR thermal profile was the same as with the original protocol, except that the annealing temperature was decreased to 38 °C to obtain better results. Subsequently, the 1st PCR products were used as templates for the 2nd PCR (tailed PCR). Using common forward and indexed reverse primers, this step permits the addition of complementary sequences for the binding sites. Products were purified, fragments in the size range of 350–800 bp were isolated, and their final concentrations were measured by quantitative PCR using Library Quantification Kit (Takara Bio, Japan) on CFX Connect™ Real-Time PCR Detection System (Bio-Rad, CA, USA). Sequencing of the multiplexed library was performed with an Illumina MiSeq Sequencer, using MiSeq Reagent Kit v3 (150 cycle, Illumina). 

The simple sequence repeat (SSR) primer region, anchors, and low-quality reads were removed using the FASTX Toolkit (http://hannonlab.cshl.edu/fastx_toolkit/). To remove the reads derived from extremely short library entries, the sequence primer region was searched in the sequences of reads 1 and 2, and the reads containing the searched sequences were removed in the TagDust software [28]. To obtain SNPs markers, pyRAD [29] was used for assembling 80-bp cleaned reads. pyRAD clustered filtered reads into loci within samples and loci into stacks between samples using VSEARCH algorithm [30], which uses measures of sequence similarity for clustering, allowing reads to contain indels. Stacks of putative orthologous loci are then aligned using MUSCLE [31]. We set the minimum depth of coverage necessary to create a stack to 6, and the similarity threshold for sequence clustering within and across samples to 0.90. Potential paralogous loci were filtered out based on the maximum number of heterozygous sites in a consensus sequences (maxH = 2) and the number of samples with shared heterozygous sites (MaxSH = 60). Further filtering was performed with TASSEL5.0 [32] to remove the samples with minimum number of missing loci >0.2, and the loci with minor allele frequency <0.03 and observed heterozygosity >0.5. The final dataset, consisting of 338 SNPs, was exported as a STRUCTURE format file (File 1), and the sequences of the loci including invariable sites were exported as a fasta file (File 2).

### 4.5. Phylogenetic Analysis Using Combined nrDNA Sequence and MIG-seq Data

We conducted maximum likelihood (ML) analyses using combined nrDNA sequences and 338 SNPs of MIG-seq data. Raw sequences of nrDNA (ITS and ETS) were assembled using BioEdit software (ver. 7.2.5 [33]). DNA sequences were aligned using MUSCLE with default settings. Alignments of ITS and ETS were combined. Gaps were deleted. We applied a hierarchical likelihood ratio test using JModeltest 2 [34,35] to determine the best-fit model of sequence evolution for both datasets in the ML analysis. For both datasets, GTR Gamma model was selected as optimum. RAxML GUI ver.2.0.0. beta 5 [36] was used to construct phylogenetic trees. Analysis was performed using a Maximum Likelihood (ML) search for 1000 rapid bootstraps. The phylogenetic tree was visualized in Figtree v1. 4 [37]. In case of the MIG-seq data of the 183 accessions, including *M. fargesii* and *M. urticifolia* as outgroup, File 2 was converted to the Phylip format using GenAlEx6.51b2 [38], and then only variable sites were extracted. The extracted SNPs data were analyzed by IQ-TREE 1.6.12 [39] to estimate an ML tree with substitution model of GTR and ascertainment bias correction (ASC) option on. A total of 1000 bootstraps were preformed to assess the clade supports. 

### 4.6. Population Genetic Analysis Using MIG-seq Data

To infer the population genetic structure of *M. montis-koyae* within each country and between the two countries, we prepared and analyzed three kinds of dataset including: (a) all individuals (11 populations, *n* = 179), (b) Japanese populations only (seven populations, *n* = 100), and (c) Chinese populations only (four populations, and four subpopulations were recognized in the Chun’an population *n* = 79). The individual-based genetic structure was estimated by Bayesian method implemented in STRUCTURE 2.3.4 [40]. For each number of groups (K) considered, which ranged from 1 to 11, we performed 10 independent Markov Chain Monte Carlo (MCMC) runs with 100,000 iterations, following a burn-in period of 10,000 steps. We determined the *K* value that best explained our data at the uppermost hierarchical level, considering the log likelihood of the data for each *K* [lnP(D)] [40] and the ad hoc statistic *ΔK* [41]. Nei unbiased genetic distances, Shannon’s Information Index, observed and expected heterozygosity for each population, were calculated based on the MIG-seq SNPs (File 1) using GenAlEx 6.51b2 [38]. 

### 4.7. Divergence Time Analysis

To estimate the divergence times of Chinese and Japanese *M. montis-koyae,* we used combined nrDNA (ITS + ETS) sequences of nine of the former and five of the latter *M. montis-koyae* samples, other *Meehania* species, and distant relatives such as *Caryopteris incana* (Houtt.) Miq. and *Melissa officinalis* L. to enable multiple fossil calibrations. In total, we used 40 taxa of which 25 were obtained from Genbank (Supplementary Table S1). 

For fossil calibration points, we used hexacolpate and three-nucleate pollen fossil from Early Eocene sediments in India identified as *Ocimum* [42], and a fruit fossil of *Melissa* from the Early-Middle Oligocene [43], following recent Lamiaceae studies [9,44,45]. For the nrDNA dataset, the Nepetoideae crown group was constrained with a lognormal prior having an offset of 49 million years (Ma), a mean of 2.6 and a standard deviation (SD) of 0.5. We also constrained the most recent common ancestor of *Melissa* L. and *Lepechinia* Willd, with a log-normal distribution having an offset of 28.4 Mya, a mean of 1.5 and SD of 0.5.

Bayesian dating based on a relaxed-clock model was used to estimate the divergence times of the main clades of *Meehania* using BEAST ver. 2.6.2 [46]. The nrDNA analyses were performed using the GTR model of nucleotide substitution. The tree prior model (birth–death) was implemented in the analysis. Posterior distributions of parameters were approximated using two independent MCMC analyses of 20,000,000 generations (sampling once every 2000 generations). Convergence of the chains was checked using Tracer 1.7 [47], and the effective sample size (ESS) was well over 200 for all categories. A maximum clade credibility tree was built using Tree Annotator [48], discarding 10% of trees as burn-in.

## Figures and Tables

**Table 1:** Pairwise Population Matrix of Nei Unbiased Genetic Distance.

	Koga-yano	Ayu	Seppi-ko	Ona-ru	Sayo	Kami-no	Shiso	Sui-chang	Song-yang	Ta-ishun	Chun’-an
**Kogayano**	0.000										
**Ayu**	0.315	0.000									
**Seppiko**	0.308	0.309	0.000								
**Onaru**	0.304	0.306	0.297	0.000							
**Sayo**	0.304	0.304	0.296	0.293	0.000						
**Kamino**	0.309	0.310	0.302	0.300	0.301	0.000					
**Shiso**	0.304	0.308	0.295	0.292	0.292	0.301	0.000				
**Suichang**	0.573	0.579	0.579	0.570	0.565	0.581	0.575	0.000			
**Songyang**	0.592	0.597	0.597	0.588	0.585	0.600	0.593	0.426	0.000		
**Taishun**	0.563	0.559	0.563	0.558	0.556	0.569	0.563	0.427	0.425	0.000	
**Chun’an**	0.563	0.563	0.564	0.560	0.557	0.571	0.565	0.415	0.432	0.406	0.000

**Table 2:** Genetic characters of 11 *Meehania montis-koyae* populations from China and Japan based on 338 SNPs by MIG-seq analysis. *I* = Shannon’s Information Index, *Ho* = observed heterozygosity, *He* = expected heterozygosity.

Pop Code		*N*	*I*	*Ho*	*He*
Kogayano	Mean	8.840	0.008	0.007	0.005
(*n* = 13)	SE	0.306	0.004	0.004	0.003
Ayu	Mean	7.805	0.012	0.007	0.007
(*n* = 13)	SE	0.288	0.004	0.003	0.003
Seppiko	Mean	9.858	0.007	0.004	0.004
(*n* = 15)	SE	0.333	0.003	0.002	0.002
Onaru	Mean	7.266	0.011	0.007	0.007
(*n* = 11)	SE	0.247	0.004	0.003	0.003
Sayo	Mean	8.308	0.011	0.008	0.007
(*n* = 13)	SE	0.288	0.004	0.003	0.003
Kamino	Mean	10.630	0.012	0.007	0.008
(*n* = 19)	SE	0.397	0.004	0.003	0.003
Shiso	Mean	11.024	0.004	0.004	0.002
(*n* = 16)	SE	0.366	0.002	0.003	0.002
Suichang	Mean	6.672	0.017	0.007	0.012
(*n* = 12)	SE	0.272	0.006	0.004	0.004
Songyang	Mean	7.855	0.014	0.004	0.009
(*n* = 16)	SE	0.355	0.005	0.002	0.003
Taishun	Mean	8.704	0.020	0.012	0.013
(*n* = 17)	SE	0.370	0.006	0.005	0.004
Chun’an	Mean	18.769	0.014	0.006	0.010
(n*n* = 33)	SE	0.743	0.005	0.003	0.003

## Supplementary Materials

The following are available online at https://www.mdpi.com/2223-7747/9/9/1159/s1, Table S1: Samples, vouchers, and accession number of ETS and ITS sequences used in this study. Accession number in bold letter indicates the sequences determined in this study.
