# Supplementary material for: A Narrow Endemic or a Species Showing Disjunct Distribution? Studies on Meehania montis-koyae Ohwi (Lamiaceae)
Source: Plants (Basel). 2020 Sep 8;9(9):1159. doi: 10.3390/plants9091159 (PMC7570357; doi:10.3390/plants9091159)
Supplement: Supplementary file 1 [file plants-09-01159-s001.pdf]

Table S1. Sambles, vouchers, and accession number of ETS and ITS sequences used in this study. Accession number in bold letter indicates the sequences determined in this study.

Taxon name	code	ETS	ITS	voucher	References
Agastache pallida (Lindl.) Cory		JQ669144	JQ669075	B.Drew 118	
Agastache rugosa Kuntze		JQ669145	JQ669076	H.Kanai, K Hasegawa, K.Ohkubo 8916	
Caryopteris incana Miq.		KY552620	EF508064	PNLI20120421-1	Li et al. 2017
Glechoma longituba (Nakai) Kuprian.		KM886687	KM886722	T.Deng 415 (KUN)	Deng et al. 2015
Glechoma biondiana (Diels) C.Y.Wu & C.Chen		KM886693	KM886728	D.G.Zhang 4446(KUN)	Deng et al. 2015
Glechoma biondiana (Diels) C.Y.Wu & C.Chen		KM886691	KM886726	D.G.Zhang 4731(KUN)	Deng et al. 2015
Glechoma biondiana v. *angustituba* C.Y.Wu & C.Chen		KM886685	KM886720	D.G.Zhang 4583(KUN)	Deng et al. 2015
Glechoma hederacea L.		JQ669171	JQ669099	B.Drew 69	
Hyptis laniflora Benth,		JF304259	JF301548	JP Lewis 2034(K)	unpublished
Lamium album L.		JX893206	JX893229	Liu and Xiang 128	
Lycopus cavaleriei H. Lév.	China	KM886695	KM886730	SNJ Exped.20110807071(KUN)	
Lycopus cavaleriei H. Lév.	Japan	LC202992	**LC542952**	J.Oda 8240(KYO)	Takano & Oda 2017, present study
Lycopus uniflorus Michx.		LC202993	**LC542953**	J.Oda & A.Muranaga 8555(KYO)	Takano & Oda 2017, present study
Lepechinialeucophylloides (Ramamoorthy, Hiriart & Medrano) B.T.Drew, Cacho & Sytsma		JF301327	JF301354	B.Drew 129	Drew & Sytsma 2011. Syst.Bot. 36: 1038-1049
Nepeta cataria L.		JQ669202	JQ669126	B.Drew 72	Drew & Sytsma 2012
Melissa officinalis L.		JF301325	JF301353	B.Drew 70	Drew & Sytsma 2011. Syst.Bot. 36: 1038-1049
Prunella vulgaris L.		JQ669206	JQ669130	J.Walker 3225	Drew & Sytsma 2012
Meehania cordata (Nutt.) Britton		JQ669187	JQ669112	A.E. Radford 45379	
Meehania urticifolia (Miq.)Makino	China	JQ669188	JQ669113	L.S.S.Hanrong	
Meehania urticifolia (Miq.)Makino	Korea	KM886713	KM886746	#39114 (AJOU)	Deng et al. 2015
Meehania urticifolia (Miq.)Makino	Japan	**LC542935**	**LC542954**	S.Fuse 2411(HYO)	Present study
Meenania faberi (Hemsl.) C.Y.Wu		KM886698	KM886733	T.Deng 438	Deng et al. 2015
Meehania fargesii (H. Lév.) C.Y.Wu var. *pedunculata* (Hemsl.) C.Y.Wu		KM886700	KM886735	D.G.Zhang 6091	Deng et al. 2015
Meehania fargesii (H. Lév.) C.Y.Wu		**LC542936**	**LC542955**	D.Takahashi s.n. (HYO)	Present study
Meehania fargesii (H. Lév.) C.Y.Wu		**LC542937**	**LC542956**	S.Sakaguchi s.n.(HYO)	Present study
Meehania fargesii var. *pinetorum* (Hand.-Mazz.)C.Y.Wu		KM886703	KM886737	C.L.Xiang 056	Deng et al. 2015
Meehania fargesii var. *pinetorum* (Hand.-Mazz.)C.Y.Wu		KM886704	KM886738	C.L.Xiang 357	Deng et al. 2015
Meehania fargesii var. *radicans* (Vaniot)C.Y.Wu		KM886704	KM886739	D.G.Zhang 6502	Deng et al. 2015
Meehania henryi (Hemsl.) Y.Z.Sun ex C.Y.Wu var. *kaitcheensis* (H. Lév.) C.Y.Wu		KM886708	KM886741	D.G.Zhang L.Xu109	Deng et al. 2015
Meehania hongliniana B.Y.Ding & X.F.Jin		**LC542938**	**LC542957**	living collection at Zhejiang BG	Present study
Meehania montis-koyae Ohwi	Suichang	**LC542939**	**LC542958**	X.-L. Xie XL170009 (HZU)	Present study
Meehania montis-koyae Ohwi	Songyang	**LC542940**	**LC542959**	X.-L. Xie XL170209 (HZU)	Present study
Meehania montis-koyae Ohwi	Taishun	**LC542941**	**LC542960**	X. Liu LP173032 (HZU)	Present study
Meehania montis-koyae Ohwi	Chun'an55	**LC542942**	**LC542961**	Z.-H. Chen LP174755 (HZU)	Present study
Meehania montis-koyae Ohwi	Chun'an58	**LC542943**	**LC542962**	Z.-H. Chen LP174758(HZU)	Present study
Meehania montis-koyae Ohwi	Chun'an	**LC542944**	**LC542963**	Xia 150521 (ZJFC)	Present study
Meehania montis-koyae Ohwi	Suichang2	**LC542945**	**LC542964**	Xia 160413 (ZJFC)	Present study
Meehania montis-koyae Ohwi	Xia 215	KM886710	KM886743	G.H.Xia 215(KUN)	Deng et al. 2015
Meehania montis-koyae Ohwi	Tonglu	**LC542946**	**LC542965**	living collection at Zhejiang BG	Present study
Meehania montis-koyae Ohwi	Sayo	**LC542947**	**LC542966**	A.Takano 090520-1(HYO)	Present study
Meehania montis-koyae Ohwi	Shiso	**LC542948**	**LC542967**	A.Takano 090520-11(HYO)	Present study
Meehania montis-koyae Ohwi	Kamino	—	—	A.Takano 090520-12(HYO)	Present study
Meehania montis-koyae Ohwi	Seppiko	**LC542949**	**LC542968**	A.Takano 090519-3(HYO)	Present study
Meehania montis-koyae Ohwi	Ayu	**LC542950**	**LC542969**	A.Takano 130531-1(HYO)	Present study
Meehania montis-koyae Ohwi	Onaru	—	—	A.Takano 090519-4(HYO)	Present study
Meehania montis-koyae Ohwi	Kogayano	**LC542951**	**LC542970**	A.Takano 090520-9(HYO)	Present study
